# P58 YOUNG PERSONS' PERSPECTIVES ON THE RESULTS OF A UK MULTI-CENTRE SURVEY ON VARIATIONS IN CLINICAL PRACTICE IN PAEDIATRIC RHEUMATOLOGY UNITS DURING THE COVID-19 PANDEMIC

**DOI:** 10.1093/rap/rkac067.058

**Published:** 2022-09-28

**Authors:** Diarmuid McLaughlin, Samundeeswari Deepak, Laura Lunt, Beth Dillon, Ecem Esen, Janet McDonagh

**Affiliations:** Department of Paediatric Rheumatology, Musgrave Park Hospital, Belfast, Northern Ireland, United Kingdom; Department of Paediatric Rheumatology, Nottingham University Hospital, Nottingham, United Kingdom; Centre for Epidemiology Versus Arthritis, Centre for Musculoskeletal Research, Manchester Academic Health Science Centre, University of Manchester and NIHR Manchester Biomedical Research Centre, Manchester University NHS Foundation Trust, Manchester, United Kingdom; Your Rheum, a national advisory group of the Barbara Ansell National Network for Adolescent Rheumatology, Manchester, United Kingdom; Your Rheum, a national advisory group of the Barbara Ansell National Network for Adolescent Rheumatology, Manchester, United Kingdom; Versus Arthritis Centre for Epidemiology; Centre for MSK Research, University of Manchester; NIHR Biomedical Research Centre, Manchester University Hospital NHS Trust; Department of Paediatric and Adolescent Rheumatology, Royal Manchester Children's Hospital, Manchester University Hospitals NHS Trust, Manchester, United Kingdom

## Abstract

**Introduction/Background:**

A paediatric rheumatology multi-centre survey was conducted to understand the impact of COVID-19 on services and provide useful learning points that could change future practice. The survey focused on clinicians' views of the pandemic. The aim of this study was to gather the perspectives of young people regarding the results of the initial survey. This was done via a group discussion with members of the national youth advisory panel for adolescent rheumatology – Your Rheum. It is important when evaluating service provision that not only the clinician’s perspective but the patient’s voice is heard.

**Description/Method:**

The initial survey was sent, in August 2021, to Consultants in all UK paediatric rheumatology centres, to the paediatric rheumatology trainees network and to the JIA topic specific group members via email. 20 staff responded (17 consultants and 3 trainees at registrar level) across the 4 UK nations. Results of the survey showed changes to the frequency of blood monitoring (n = 17), an increase in disease flares (n = 4) with all respondents reporting changes to the type of consultation used with a large increase in the use of virtual consultations. Increases in clinic waiting lists due to capacity issues were also reported (n = 7). 15 respondents reported changes in steroid usage for flare management with more ward based steroid injections performed and an increase in the use of oral steroids due to theatre unavailability.

The online discussion group held with ‘Your Rheum’ members consisted of 9 Young People (YP) aged between 12-24 years. The discussion group was facilitated by a research assistant, paediatric rheumatology trainee and a consultant paediatric rheumatologist. A number of statements with Agree/Disagree/Unsure options were provided with an opportunity to vote on each statement. A follow up discussion was held after each statement around the issues raised. The online tool Mentimeter was used to form a word cloud on YP experiences of the pandemic which was followed by breakout rooms to facilitate further small group discussions.

**Discussion/Results:**

In contrast to the survey results which revealed that clinicians perceived that YP preferred virtual (telephone or video or combination of both) consultations, young people in ‘Your Rheum’ varied in their responses with the majority of comments describing that it depended on the clinical context (if unstable or stable disease) with virtual appointments being preferable when their disease was stable. YP also reported issues with virtual appointments including difficulties with phone signal, not being able to obtain a specific time for appointments (varied time slots), issues for YP with hearing difficulties as well as one YP commenting it is “easier to lie to the doctor and just say everything is going really well”. Negative points raised with face-face appointments included longer waiting times and the time spent travelling to appointments.

The majority of young people agreed that changes to the normal pattern of blood monitoring was concerning as this could lead to a possible increase in flare of conditions. Specific comments including accessibility to appointments was difficult during the pandemic with the additional fear of getting COVID-19 if attending the hospital for blood monitoring.

YP used a variety of words to explain their experiences of rheumatology services during the pandemic including: ‘rushed’, ‘frustrating’, ‘stressful’, ‘virtual’, ‘chaotic’, ‘poor communication’, ‘inaccessible’ but also ‘COVID safe’.

**Key learning points/Conclusion:**

Young people with rheumatic diseases offered different insights to the initial clinician survey findings highlighting the importance of considering their opinion as well as that of professionals when considering service provision and evaluation.

Flexibility is essential for young people and a hybrid approach with virtual/face to face appointments is key. Overall YP prefer face-face but are willing to have virtual consultations depending on the nature of issues needing addressed and the stability of disease.

Experiences of poor communication between YP and health professionals/hospital has always been a theme in their experiences to date but has worsened since COVID-19.

YP within this group discussion preferred to have blood monitoring performed at regular frequencies (similar to pre-pandemic) but did have concerns about getting COVID-19 by attending these appointments during the pandemic. It is important that YP are encouraged and reassured about attending appointments whilst ensuring COVID-19 safe precautions.

